# An adeno-associated viral vector transduces the rat hypothalamus and amygdala more efficient than a lentiviral vector

**DOI:** 10.1186/1471-2202-11-81

**Published:** 2010-07-13

**Authors:** Marijke WA de Backer, Carlos P Fitzsimons, Maike AD Brans, Mieneke CM Luijendijk, Keith M Garner, Erno Vreugdenhil, Roger AH Adan

**Affiliations:** 1 Rudolf Magnus Institute of Neuroscience, Department of Neuroscience and Pharmacology, University Medical Centre Utrecht, Utrecht, the Netherlands; 2 Medical Pharmacology Department, Leiden/Amsterdam Center for Drug Research, Leiden University Medical Center, Leiden University, Leiden, the Netherlands

## Abstract

**Background:**

This study compared the transduction efficiencies of an adeno-associated viral (AAV) vector, which was pseudotyped with an AAV1 capsid and encoded the green fluorescent protein (GFP), with a lentiviral (LV) vector, which was pseudotyped with a VSV-G envelop and encoded the discosoma red fluorescent protein (dsRed), to investigate which viral vector transduced the lateral hypothalamus or the amygdala more efficiently. The LV-dsRed and AAV1-GFP vector were mixed and injected into the lateral hypothalamus or into the amygdala of adult rats. The titers that were injected were 1 × 10^8 ^or 1 × 10^9 ^genomic copies of AAV1-GFP and 1 × 10^5 ^transducing units of LV-dsRed.

**Results:**

Immunostaining for GFP and dsRed showed that AAV1-GFP transduced significantly more cells than LV-dsRed in both the lateral hypothalamus and the amygdala. In addition, the number of LV particles that were injected can not easily be increased, while the number of AAV1 particles can be increased easily with a factor 100 to 1000. Both viral vectors appear to predominantly transduce neurons.

**Conclusions:**

This study showed that AAV1 vectors are better tools to overexpress or knockdown genes in the lateral hypothalamus and amygdala of adult rats, since more cells can be transduced with AAV1 than with LV vectors and the titer of AAV1 vectors can easily be increased to transduce the area of interest.

## Background

Viral vectors are used as tools to introduce genes or short-hairpin RNAs (shRNAs) into the brain in order to unravel the role of genes. The advantages of viral vectors are that they can be injected locally and that they establish long term expression of a gene or shRNA. Several viral vectors have been tested *in vivo *in the central nervous system, such as adeno-associated viral (AAV), lentiviral (LV), adenoviral (AdV) and herpes simplex viral (HSV) vectors [1-4]. To date, studies in the rodent hypothalamus and amygdala mainly have used AAV or AdV and to a lesser extent LV vectors [5-10]. In this study we compared the transduction efficiencies of AAV and LV vectors. The LV and AAV vector used in this study both used the CMV promoter to drive the expression of a fluorescent marker, dsRed or GFP respectively. The AAV and LV vectors were pseudotyped; the AAV vector was pseudotyped with AAV1 and the LV vector was pseudotyped with VSV-G. To date, at least 12 serotypes of AAV are discovered (AAV1-AAV12) [11-20] that probably use different receptors to enter cells [21-28]. Nevertheless, for most serotypes the cell entry receptors are still unknown. Until now the most widely used serotype is AAV2. However, recent studies have shown that AAV1 and AAV8 coated vectors transduce more neurons than AAV2 coated vectors *in vivo *in several brain areas, such as the rat striatum, hippocampus, midbrain [29-35]. We have chosen to use an AAV1 pseudotyped vector, because we previously have shown that this serotype is more efficient in transduction of neurons in the adult rat hypothalamus than an AAV2 encapsidated vector [36]. For the LV vector we chose the vesicular stomatitis virus glycoprotein (VSV-G), because it was shown to have a broad tropism for all kinds of neurons. However there are also other envelope proteins which can be used to pseudotype LV vectors and target the CNS [37].

It is important to know which viral vector, AAV or LV, is most efficient in transduction of brain areas involved in energy homeostasis, because then it is possible to efficiently alter gene expression in specific brain nuclei to further investigate the function of genes involved in feeding behavior. The lateral hypothalamus (LH) and amygdala (AM) are important brain areas involved in energy homeostasis [38]. Previously, we have shown that AAV vectors can be used to alter behavior in rats. AAV-mediated overexpression of neuropeptide Y, agouti and agouti-related peptide in the hypothalamus increased parameters such as body weight and food intake [36,39-41]. However, a study by another group showed that LV vectors can be also used to alter gene expression in the hypothalamus and thereby alter body weight [42]. Thus, AAV and LV vectors are able to change gene expression and behavior after transduction of neurons in the rat hypothalamus. However, it is unclear whether AAV or LV vectors are more efficient in transduction of the rat hypothalamus or amygdala. Therefore, this study compared the transduction efficiencies of a LV and an AAV vector in the LH and AM of adult rats.

## Results

### *In vitro *testing of antibodies

**Figure 1:**
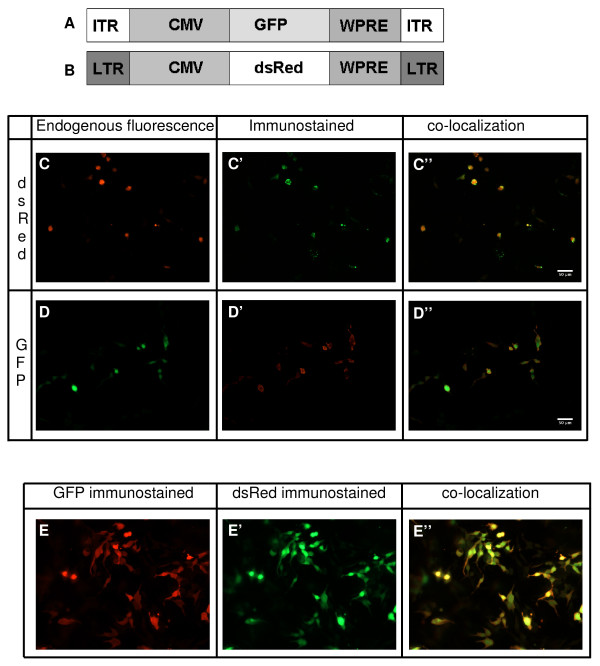
***In vitro *testing of the antibodies against GFP and dsred**. **A**: schematic overview of AAV vector used. **B**: schematic overview of LV vector used. **C**: shows endogenous dsRed fluorescence (red) in 293T cells transfected with CMV-dsRed. **C'**:shows immunostaining for dsRed (green) in these cells. **C''**: shows the co-localization of the immunostained and endogenous fluorescence (yellow). **D**: shows endogenous fluorescence (green) in 293T cells transfected with CMV-GFP. **D'**: shows immunostaining for GFP (red) and **D'' **shows the overlay of D and D' (green nucleus with red cytoplasma). **E**: 293T cells co-transfected with CMV-GFP and CMV-dsred were immunostained for GFP (red (**E**)) and dsRed (green (**E'**)). These stainings overlap (yellow (**E''**)). Scalebar is 50 μm.

To confirm that GFP and dsRed antibodies were able to detect the respective proteins with similar efficiencies, 293T cells were transfected with constructs encoding for CMV-GFP and/or CMV-dsRed (Figure 1A, B). Since the promoter driving the expression of the fluorescent proteins was the same, we expected similar levels of expression. The endogenous fluorescence of GFP and dsRed was compared with the immunostained fluorescence. The cells transfected with only one construct showed co-localization of endogenous fluorescence and immunostained fluorescence (Figure 1C, D). In addition, cells co-transfected with CMV-GFP and CMV-dsRed showed co-localization of both immunohistochemistry signals (Figure 1E). The pictures showed that red fluorescent signals, endogenous or immunostained, were at least threefold stronger in intensity than green fluorescent signals.

### AAV1-GFP and LV-dsRed transduction *in vitro*

The AAV-GFP plasmid was pseudotyped with an AAV1 coat, because that coat was previously shown to be more effective in transduction of hypothalamic nuclei, such as the LH, than AAV2 coated vectors [36]. It is still unknown which entry receptors AAV1 uses to enter cells and it is therefore unknown which cell line is most optimal for determining transducing units. Nevertheless, we performed a serial dilution with AAV1-GFP on HT-1080 cells to obtain an indication of transducing units. These results showed that the titer of AAV1-GFP was 5 × 10^8 ^t.u./ml, which is substantially lower than the 6.6 × 10^13 ^g.c./ml. The LV-dsRed had a titer of 3.9 × 10^8 ^t.u./ml.

Before mixing the two viruses, the preparations were diluted. AAV was diluted to 2 × 10^8 ^and 2 × 10^9 ^g.c./μl, thus 1.5 × 10^3 ^and 1.5 × 10^4 ^t.u./μl on HT-1080 respectively. LV-dsRed was diluted to 2 × 10^5 ^t.u./μl. Subsequently the diluted viruses were mixed 1:1 and 1 μl of this mix was injected in each brain area. This resulted in 1 × 10^8 ^or 1 × 10^9 ^g.c. (7.5 × 10^2 ^or 7.5 × 10^3 ^t.u.) of AAV1-GFP and 1 × 10^5 ^t.u. of LV-dsRed per site.

### Transduction of the LH by AAV1-GFP and LV-dsRed

**Figure 2:**
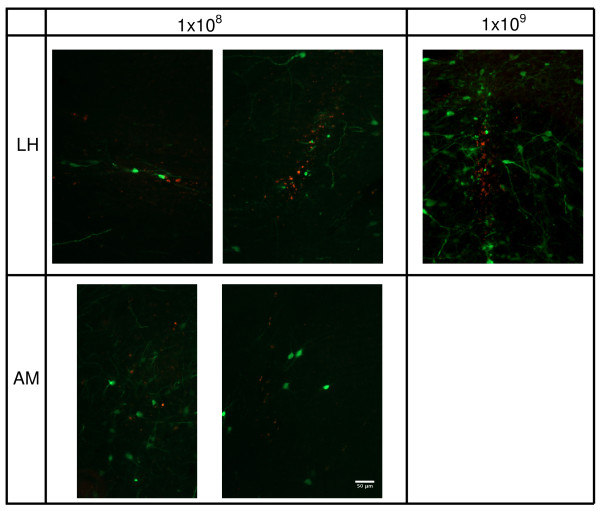
***In vivo *transduction of the LH and AM by AAV1-GFP and LV-dsRed**. The titer of LV-dsRed was kept constant at 1 × 10^5 ^t.u., while the titer of AAV1-GFP was 1 × 10^8 ^or 1 × 10^9 ^g.c. Immunostaining for GFP is shown in green and for dsRed in red.

**Figure 3:**
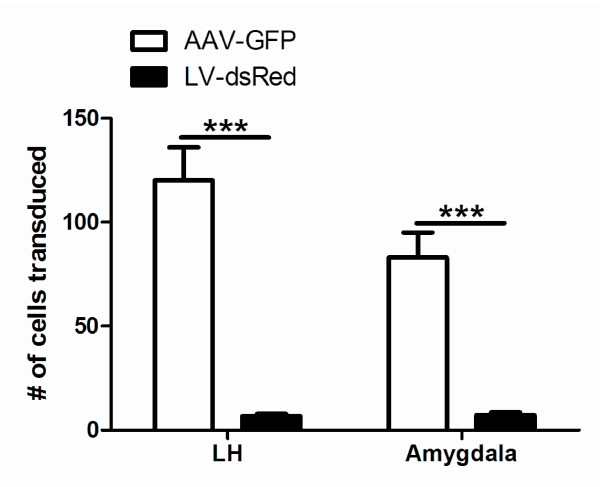
**Quantification of the total number of cells transduced AAV1-GFP and LV-dsRed**. Graphical representation of the total number of cells which where transduced by 1 × 10^8 ^g.c. of AAV1-GFP and by 1 × 10^5 ^t.u. of LV-dsRed in the LH or in the AM. *** p < 0.0001.

**Figure 4:**
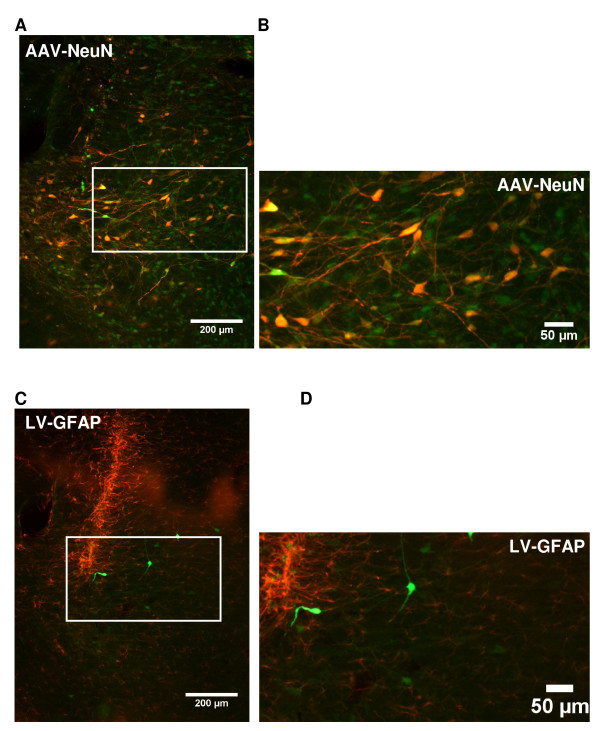
**Co-localization of GFP and NeuN and dsRed and GFAP in the LH and AM**. **A**: shows that AAV1-GFP (red, 1 × 10^9^) and NeuN (green) co-localize in the LH. **B**: shows a close-up of AA1V-GFP and NeuN co-localization. **C**: shows no co-localization of dsRed (green) and GFAP (red) in the LH. **D**: shows no overlap between LV-red (green) and GFAP (red).

To determine the transduction efficiencies of AAV and LV vectors in the LH the animals were perfused four weeks after injection with viral vectors. Immunostaining for GFP and dsRed showed positive staining in the injection tract. These were probably apoptotic cells and were not included in our quantification (Figure 2, upper panel). Counting of all GFP and dsRed immunostained positive cells revealed that AAV1-GFP, at 1 × 10^8 ^g.c., transduced significantly more cells in the LH compared with LV-GFP, at 1 × 10^5 ^t.u. (Figure 3). The total area transduced by AAV1-GFP at the injection site was 3338 (± 599) μm^2 ^and an area of 2441 (± 403) μm^2 ^was transduced by LV-dsRed. As expected, an increase in titer of AAV1-GFP from 1 × 10^8 ^to 1 × 10^9 ^g.c. in the LH resulted in 14.2-fold increase in the area transduced at the injection site; the total transduced area at the injection site increased from 3338 (± 599) μm^2 ^to 47525 (± 10822) μm^2 ^(*p *= 0.0005). In the LH AAV1-GFP predominantly transduced neurons, because GFP and NeuN co-localize (Figure 4A, B) and LV-dsRed probably also transduced mainly neurons, because dsRed and GFAP did not co-localize (Figure 4C, D).

### Transduction of the AM by AAV1-GFP and LV-dsRed

In addition, we studied the transduction efficiencies of AAV1-GFP and LV-dsRed in the AM (Figure 2, lower panel). Similar to the LH, AAV1-GFP transduced significantly more cells than LV-dsRed in the AM (Figure 3**)**. The number of cells transduced by AAV1 or LV in the AM was comparable to the numbers transduced in the LH. In the AM AAV1 and LV vector also predominantly transduced neurons, however some cells transduced with AAV1-GFP did not co-localize with NeuN and had a microglia appearance.

## Discussion

This study showed that an AAV1 vector transduced significantly more cells in the LH and AM of rats than a LV-VSV-G vector. This is in agreement previous studies which showed low levels of transgene expression after injection of 1 μl of LV vectors in other rat brain nuclei, namely the red nucleus [43] and the retina [44]. In contrast, several studies reported substantial levels of transduction by LV in the rat striatum and hippocampus, however, these groups injected larger volumes of LV, namely 2 or 3 μl [45-47]. In addition, LV vectors have been reported to efficiently transduce cells in the mouse hippocampus and striatum [48,49] and AAV and LV vectors were reported to transduce approximately similar numbers of cells after injection in mouse hippocampus or hippocampal slices [50,51]. These data indicate that there may be species differences and/or brain area differences, which may contribute to variations in transduction efficiencies by LV and AAV vectors in rat and mouse brain.

The titer of our AAV1-GFP preparation was 6.6 × 10^13 ^g.c./ml, which is a titer in the range that we usually obtain with AAV production. The number of transducing units/ml will be lower than genomic copies/ml since not all vector DNA is properly packaged into infectious particles. The optimal cell line for determining the t.u./ml of AAV1 preparations is unknown. We assessed the t.u./ml of AAV1-GFP on HT-1080 cell line. The titer of AAV1-GFP was 5 × 10^8 ^t.u./ml, and this prep was diluted 330 times, to 1.3 × 10^3 ^t.u./μl before mixing with LV-dsRed. The LV-dsRed was only diluted 2 times to 2 × 10^5 ^t.u./μl. Since the number of t.u. of AAV1-GFP injected was much lower than the t.u. of LV-dsRed, we conclude that AAV1 is more efficient in transduction of the LH and AM than LV-VSV-G. However, it has to be kept in mind that comparison of t.u. of LV-VSV-G and AAV1 vector preparations on cell lines is complicated, because the vectors probably use different, still unknown, entry receptors and different cell lines have different surface receptors. For example, one dose of AAV1/2 vectors has different t.u. values on different cell lines [52]. In addition, the t.u. of LV-dsRed preparation was assessed in the presence of polybrene, which enhances transduction [53], however, polybrene was not added when LV-dsRed was injected *in vivo*.

In the LH AAV1-GFP at 1 × 10^8 ^g.c. al ready transduced more cells than LV-dsRed. When the titer of AAV1-GFP was increased a ten-fold, the area transduced and the numbers of neurons transduced were increased accordingly. This indicates that increasing the titer is a valid method to increase the number of transduced neurons.

We only observed 6-7 dsRed positive cells after injection of 1 × 10^5 ^t.u. of LV. Thus, ideally the titer of LV-dsRed should be increased to transduce more cells in the LH and AM. The methods we used for LV preparation and titer determination are standard procedures in the field. Normally, titers of concentrated LV vectors are reported to be in the range of 5 × 10^7 ^to 1 × 10^9 ^t.u./ml [45,48,54]. Thus compared with results from others, 3.9 × 10^8 ^t.u./ml is a high titer. Therefore, it is technically difficult to increase the LV titer to transduce more neurons in the AM and LH of rats.

The differences in transduction by AAV1-GFP and LV-dsRed in the LH and AM may be explained by the fact that the vectors probably used different receptors to enter cells. The LV-dsRed used in this study was pseudotyped with VSV-G. VSV-G transduces many cell types from different species, but it is still unknown how VSV-G enters these cells. For a long time it was thought that phosphatidylserine (PS) was the receptor to mediate membrane fusion [55]. However, more recent data indicated that PS is not the entry receptor [56]. AAV-GFP was pseudotyped with AAV1 capsid proteins. The entry receptor for AAV1 also is unknown, but there are indications that α-2,3 and α-2,6 N-linked sialic acids facilitate transduction by AAV1 vectors [23].

Exchanging GFP and dsRed genes between LV and AAV vector (thereby obtaining AAV-dsRed and LV-GFP) probably will not alter the transduction efficiencies which we observed in this study, since the promoter and other parts of the vectors including the viral coat are unchanged. Previous studies have compared expression of GFP and dsRed in LV or AAV vectors and showed that the expression profile remained the same when only the fluorescent markers were changed; co-injection of two vectors (e.g. LV-GFP and LV-dsRed) showed that expression of both vectors largely overlapped [45,57].

The co-localization of AAV1 and NeuN showed that AAV1 predominantly transduced neurons in the LH and AM. This is in agreement with previous studies which showed that AAV1 predominantly transduced neurons in different rat brain regions at all investigated time points after injection [29,33,34]. The LV and GFAP immunohistochemistry signals did not co-localize indicating that LV at 4 weeks post-injection probably transduced neurons. This confirms data from previous studies where LV predominantly co-localized with NeuN in the rat brain [45,46,58,59].

## Conclusion

When a substantial part or the entire LH or AM of rats needs to be transduced AAV1 vectors are preferred over LV vectors, since more neurons can be transduced with AAV1 than with LV-VSV-G vectors and the titer of AAV preparations can easily be increased.

## Methods

### Cell lines and constructs

Human embryonic kidney (HEK) 293T cells were maintained at 37°C with 5% CO_2 _in Dulbecco's modified Eagles medium (DMEM) supplemented with 10% fetal calf serum (FCS), 2 mM glutamine, 100 units/ml penicillin, 100 units/ml streptomycin and non-essential amino acids.

pAAV-CMV-GFP was constructed by removing the CMV promoter with a part of the GFP gene from pTRCGW [60] through digestion with KpnI and BsrGI. Subsequently this fragment was ligated into a KpnI BsrGI digested backbone of pAAV-CBA-GFP (kind gift from M. Sena-esteves [61]). This digestion removed the CBA promoter and a part of the GFP gene.

The construction of LV-CMV-dsRED was previously described [62].

To check the specificity and intensity of staining of the GFP and dsRed antibodies, 2.5 μg of pPRIME-CMV-GFP and pPRIME-CMV-dsRed [63] were transfected, alone or together, on 10 cm dishes with polyethylenimine (PEI). The morning after transfection the cells were trypsinized and seeded in 24 wells plates containing poly-L-lysine coated glass cover slips. Seventy-two hours after transfections cells were washed with phosphate buffered saline (PBS), fixated for 20 minutes with 4% paraformaldehyde (PFA) and stored in PBS at 4°C until immunohistochemistry was performed.

### Virus production and purification

AAV production was performed with 15 × 15 cm dishes 293T cells, which were 80-90% confluent at day of transfection. Two hours before transfection, the 10% FCS-DMEM was replaced with 2% FCS-DMEM. The transfections were performed with polyethylenimine (PEI) as described by Reed S.E. et al. [64]. pAAV-CMV-GFP was co-transfected with the helper plasmid pDP1 [65] (Plasmid factory, Bielefeld, Germany) in a molar ratio of 1:1. The transfection mix remained on the cells until the next day, then the 2% FCS-DMEM was refreshed. The production and purification was essentially performed as described by Zolotukhin et al. [66]. Briefly, sixty hours after transfection, the cells were harvested in their medium, centrifuged and washed with PBS containing 5 mM ethylenediaminetetraacetic acid (EDTA). Finally, the cells were collected in 12 ml ice cold buffer (150 mM sodium chloride (NaCl), 50 mM 2-amino-(hydroxymethyl)-1,3-propanediol (Tris), pH 8.4 ) and stored at -20°C until further use. Subsequently, the cells were freeze-thawed twice, incubated for 30 minutes with 50 units/ml Benzonase (Sigma, the Netherlands) at 37°C and centrifuged. After centrifugation, the supernatant was loaded onto an iodixanol gradient (60%, 40%, 25%, 15%, supernatant (Optiprep, Lucron bioproducts, Belgium)) in quickseal tubes (Beckman Coulter, The Netherlands). After 1.25 hour of ultracentrifugation (70.000 rpm at 18°C) in Ti70 rotor (Beckman Coulter, the Netherlands), the 40% layer was extracted. This 40% layer was used for ion-exchange chromatography with 5 ml Hitrap Q HP columns (GE Healthcare, The Netherlands). Subsequently PCR was used to determine AAV positive fractions. The positive fractions were pooled and desalted/concentrated on Centricon Plus-20 Biomax-100 concentrator columns (Millipore, The Netherlands). The titer, in genomic copies per ml (g.c./ml), was determined by qPCR with sybergreen mix in a LightCylcer (Roche) [67]. The qPCR primers were designed to detect BGHpolyA and were BGHpolyA_F: 5' CCTCGACTGTGCCTTCTAG; BGHpolyA_R: 5' CCCCAGAATAGAATGACACCTA. The titer obtained for AAV1-GFP was 6.6 × 10^13 ^genomic copies (g.c.)/ml. In addition we also performed a serial dilution with AAV1-GFP virus on HT1080 cells, to obtain an indication of the transducing units in this AAV preparation. Seventy-two hours after infection GFP positive cells were counted and a titer was calculated. This serial dilution showed that a titer of 5 × 10^8 ^transducing units (t.u.)/ml was achieved.

Lentivirus with CMV promoter driving dsRed expression (LV-dsRED) was produced as described previously [62]. In short, 293T cells were transfected using the ViraPower Lentiviral Expression System (Invitrogen, Breda, the Netherlands) according to manufacturer's instructions. Forty-eight hours after transfection virus containing supernatant was harvested, centrifuged at 2.000 rpm for 3 minutes to remove cell debris and concentrated by two rounds of ultracentrifugation (19.400 rpm, 4°C, 2 hours each), resuspended in PBS, aliquoted and stored at -80°C until use. Virion titers were measured by real-time PCR and titers were calculated from those and verified by dsRed expression in 293T cell with addition of polybrene [68]. Titer of LV-dsRed was 3.9 × 10^8 ^t.u/ml.

### Animals

Twelve male Wistar rats of 220-250 g, were purchased from Charles River (Crl-Wu, Germany). All rats were individually housed in filtertop cages with *ad libitum *access to food (CRM pellets; Special Diet Services, Whitham, Essex, UK) and water. Animals were kept in a temperature- and humidity-controlled room (21 ± 2°C) with a 12 h light/dark cycle (lights on at 7:00 A.M.). All experimental procedures were approved by the Committee for Animal Experimentation of the University of Utrecht (Utrecht, The Netherlands).

Just before stereotactic injections AAV1-GFP was diluted in PBS to 2 × 10^8 ^g.c/μl or 2 × 10^9 ^g.c./μl and LV-dsRed was diluted to 2 × 10^8 ^tu/μl. The diluted AAV and LV vectors were mixed 1:1 and 1 μl of this mixture was injected in the LH or in the AM of rats. The injections were performed with a micro-infusion pump. The injection speed was 0.2 μl/minute. After the injection the needle remained in the injection site for 10 minutes.

After 11/2 week of acclimatization surgery was performed under fentanyl/fluanisone (Hypnorm^®^, Janssen Pharmaceutica, Beerse, Belgium, 0.1 ml/100 g intramuscular) and midazolam (Dormicum^®^, Roche, Woerden, the Netherlands, 0.05 ml/100 g intraperitonal) anesthesia. Carprofen (Rimadyl^®^, Pfizer Animal Health, Capelle a/d Ijssel, the Netherlands, 0.01 ml/100 g s.c.). Four rats were injected bilaterally in the LH (coordinates AP-2.6, ML+2.0, DV-8.6) with 1 μl of AAV-LV mixture containing 1 × 10^8 ^gc of AAV1-GFP and 1 × 10^8 ^tu of LV-dsRed. Another four rats also received 1 μl AAV-LV mixture in the LH. Here LV-dsRed remained 1 × 10^8 ^tu, however, the titer of AAV1-GFP was raised 10 times to 1 × 10^9 ^gc. In addition, four rats received injections in the central AM (coordinates AP-2.1, ML+4.0, DV -8.0) with a mixture of 1 × 10^8 ^gc of AAV1-GFP and 1 × 10^8 ^tu of LV-dsRed. Four weeks after the injections the rats were anesthetized and perfused with 4% PFA containing 0.05% glutaraldehyde. Subsequently, the brains were isolated and placed in 4%PFA overnight at 4°C. The next morning the brains were placed in PBS and stored at 4°C until further use. The perfused brains were sectioned on a vibratom (Leica) at 40 μm in series of 10.

### Immunohistochemistry

Every tenth 40 μm section was used for GFP-dsRed staining. The free floating sections were washed 3 times with PBS, permeabilized for 30 minutes in PBS supplemented with 0.5% triton X-100 at room temperature (RT), blocked for 1 hour in PBS with 1.5% normal goat serum (NGS) at RT and incubated overnight in PBS supplemented with mouse monoclonal anti-dsRed (1:400, Clontech), rabbit polyclonal anti-GFP (1:1000, Invitrogen) and 1.5% NGS at 4°C. The next morning sections were washed 3 times for 10 minutes with PBS and incubated for 1 hour with secondary antibodies (ALEXA 555 conjugated goat anti mouse (1:500) and ALEXA 488 conjugated goat anti rabbit (1:1000) both Invitrogen) in 1.5% NGS at RT. After 3 times 10 minutes wash with PBS, the sections were transferred to microscope slides and kept over night in the dark to dry. All sections were embedded in 90% glycerol and stored flat at 4°C.

A similar protocol was used for GFP-NeuN double staining, the antibody of mouse NeuN (Chemicon) was used at 1:2000 dilution and goat anti-mouse ALEXA 555 was used at 1:1000 dilution.

For dsRed-GFAP staining the sections were washed 3 × in PBS and incubated in sodium citrate buffer (10 mM tri-sodium citrate, pH = 8.5) for 30 minutes at 70°C. Subsequently, the sections were allowed to cool down to room temperature, washed 3 times for 5 minutes with PBS, blocked in PBS+ 3% fetal calf serum (FCS) for 60 minutes and incubated overnight in PBS supplemented with mouse anti-dsRed (1:500), rabbit anti-GFAP (1:4000, DAKO), 0.2% triton X-100 and 1% FCS at 4°C. The next morning the sections were washed 3 times for 10 minutes in PBS and incubated in PBS supplemented with goat anti-rabbit-ALEXA 488 (1:250), goat anti-mouse-ALEXA 555 (1:500), 0.2% triton-X100 and 1% FCS for 1 hour. After 3 times 10 minutes wash with PBS, the sections were transferred to microscope slides and kept over night in the dark to dry. All sections were embedded in 90% glycerol and stored flat at 4°C.

### Imaging and data analysis

The number of positive cells for GFP and dsRed were counted in every section, except the positive cells in the injection tract which are probably macrophages.

The MCID system was used to digitize pictures from sections containing endogenous or immunohistochemistry signals.

GraphPad Prism was used for data analysis and treatment effects were evaluated with two-tailed *t*-test.

## Competing interests

The authors declare that they have no competing interests.

## Authors' contributions

MdB constructed the AAV vector and produced the AAV vector with the help of KG. MB and ML performed animal work including the stereotaxic injections. MdB performed and analyzed immunohistochemstry; discussed the results and prepared the manuscript. CF constructed and produced the LV vector preparation; discussed and corrected the manuscript. EV participated in the design of the study and discussed and corrected the manuscript. RA participated in experimental design and supervised the experiments, discussed results, corrected the manuscript and provided financial support. All authors have read and approved the final manuscript.
